# Neuropsychiatric Symptoms and Their Association With Sex, Age, and Enzyme Replacement Therapy in Fabry Disease: A Systematic Review

**DOI:** 10.3389/fpsyt.2022.829128

**Published:** 2022-03-16

**Authors:** Magdalena Mroczek, Ignazio Maniscalco, Manon Sendel, Ralf Baron, Erich Seifritz, Albina Nowak

**Affiliations:** ^1^Department of Neurology and Neurophysiology, Balgrist University Hospital, University of Zurich, Zurich, Switzerland; ^2^Department of Geriatric Psychiatry, Psychiatric Hospital of University of Zurich, Zurich, Switzerland; ^3^Department of Neurology, Division of Neurological Pain Research and Therapy, University Clinic Schleswig-Holstein, Kiel, Germany; ^4^Department of Psychiatry, Psychotherapy and Psychosomatics, Psychiatric Hospital of University of Zurich, Zurich, Switzerland; ^5^Department of Internal Medicine, Psychiatric Hospital of the University of Zurich, Zurich, Switzerland; ^6^Department of Endocrinology and Clinical Nutrition, University Hospital Zurich, Zurich, Switzerland

**Keywords:** fabry disease, fd, cognitive impaiment, depression, ERT (enzyme replacement therapy)

## Abstract

Patients suffering from Fabry disease (FD) have an increased risk of developing neuropsychiatric symptoms (NPS), mostly impairment in cognitive performance and depression. Single cases of psychosis have been reported, however, their association with FD can be coincidental. Furthermore, deficits in social functioning and adaptation as well as specific coping styles in FD patients were observed. Recent studies focused on a longitudinal course of the disease and identified risk factors associated with specific NPS. Since 2001, enzyme replacement therapy (ERT) has been available and in preliminary studies seems to improve cognitive impairment and adaptive skills. In this systematic review, we analyze the available literature on the NPS in FD and investigate if there are any differences in their distribution between males and females, children/adolescents and adults, and individuals treated with ERT and untreated. We discuss the role of the psychological, environmental, and molecular alterations and their correlation to psychiatric manifestations in FD. Finally, we would like to increase awareness of the spectrum of NPS in FD.

## Introduction

Fabry disease (FD) is an inherited X-linked lysosomal storage disorder. FD is caused by mutations in the *GLA* (alpha galactosidase A) gene, that encodes for the lysosomal enzyme α-galactosidase A (AGA) and is located on the chromosome X (1–4). The disease affects both males and females. The resulting AGA deficiency causes a progressive accumulation of compounds with terminal α-galactosyl moieties: globotriaosylceramides and other glycosphingolipids. They accumulate in various organs in the body, including brain, kidneys and cerebral vessels, leading to the clinical manifestations of FD (5–7). The central nervous system symptoms have been described in both hemizygous (8) and heterozygous (9) individuals. The onset of the symptoms may be sudden or gradual, the disease course progressive or fluctuating.

The key component in the central nervous system (CNS) is vascular with transient ischemic attacks, cerebral hemorrhages, thrombosis, and lacunar infarcts (10, 11). Glycosphingolipid globotriaosylceramide (Gb3/GL-3) accumulates mainly in the vascular endothelium and the smooth muscle of the brain vessels (12). The white matter changes are usually secondary to vascular alterations. However, it has been suggested that a direct accumulation of Gb3 in the anatomical structures related to the occurrence of neuropsychiatric symptoms may also take place. Brain tissue deposition resulting in the neuronal ballooning and gliosis in dorsal motor nucleus of the vagus, substantia nigra, neocortex, hippocampus and brainstem, amygdala, hypothalamus and entorhinal cortex has been reported (13–15).

Several studies described depression and cognitive impairment as the main NPS in FD (16–20). Vasculopathy and micro strokes were identified as the key factors contributing to the cognitive decline (10). Although depression was previously associated prevalently with pain and worse quality of life (21, 22), other mechanisms, such as vasculopathy and secondary depression after stroke also play an important role (10). Recently, newer techniques enabled researchers to investigate NPS not only at the clinical level but also to explore their imaging and molecular correlates. The underlying mechanisms, such as activation of nitric oxide pathways, oxidative stress, and activation of prothrombotic factors have been reported (23). Glycosphingolipids accumulation in the vessels and directly in the brain tissue contributes to the psychiatric manifestations of disease and explain, at least partially, the broad spectrum of phenotypes (10, 13, 15). Applying enzyme replacement therapy (ERT) seems to have a positive influence on cognitive and psychiatric symptoms, especially in the pediatric cohorts (24, 25). In the adult cohorts, data on ERT impact on NPS are still insufficient. Other factors, such as female sex or age can also influence the development and severity of NPS in FD. Available studies showed some discrepancies that may be result of the different methodology or small cohort size.

In this manuscript we characterize NPS in FD and provide an update of the available literature. For this purpose, we reviewed articles related to NPS in FD: especially cognition and depression, but also psychosis and personality features, and analyze sex, age and therapy status as factors impacting NPS. Also, we explore NPS in specific cohorts: pediatric vs. adult, males vs. females, and the impact of ERT initiation on the development and clinical course of NPS. Finally, we discuss the role of several psychological factors on NPS.

## Materials and Methods

### Research Problem

To formulate a research questions a PICO framework has been used (26). Our primary aim was to review an available literature on NPS in FD and to describe the spectrum and studies on NPS in FD. Our secondary aim was to investigate the impact of sex, age and ERT therapy on NPS in children vs. adults, males vs. females and ERT treated individuals vs. untreated and compare them with studies on mixed, untreated FD cohorts.

### Eligibility Criteria

We applied PRISMA 2020 guidelines (27). To review the NPS in FD on 16 July 2021, PubMed database has been searched using the following criteria: (”Fabry disease” OR “X-linked lysosomal storage” OR “alpha-galactosidase” OR “globotriaosylceramide” OR “GLA gene”) AND (neurocognitive OR neurodegenerative OR psychiatry OR psychiatric OR neuropsychiatric OR neuropsychiatry OR suicide OR parasuicide OR suicidal OR parasuicidal OR psychosis OR psychotic OR dementia OR neuropsychological OR depression OR depressive OR psychosocial OR “cognitive impairment” OR “Lewy body”). Two researchers (MI, MM) independently manually reviewed titles, abstracts and full-text of all records and decided on the study inclusion. In case of inconsistencies, a consensus was reached by discussion.

In the first search run, we have identified 219 articles of interest that we further analyzed individually. We included case reports and original studies. All reviews and systematic reviews (*n* = 47) as well as meta-analysis (*n* = 2) and commentaries (*n* = 1) were excluded. Six articles were excluded because they were not written in English. We included only studies that primarily described NPS in FD. Studies primarily on pain and quality of life were excluded. Altogether, 136 articles were excluded as not suitable for the topic of this review. After inclusion, manuscripts have been assessed in terms of symptoms in males vs. females, children/adolescents and ERT + and compared with the mixed and adult FD cohorts or age- and sex-matched healthy controls.

## Results

**FIGURE 1:**
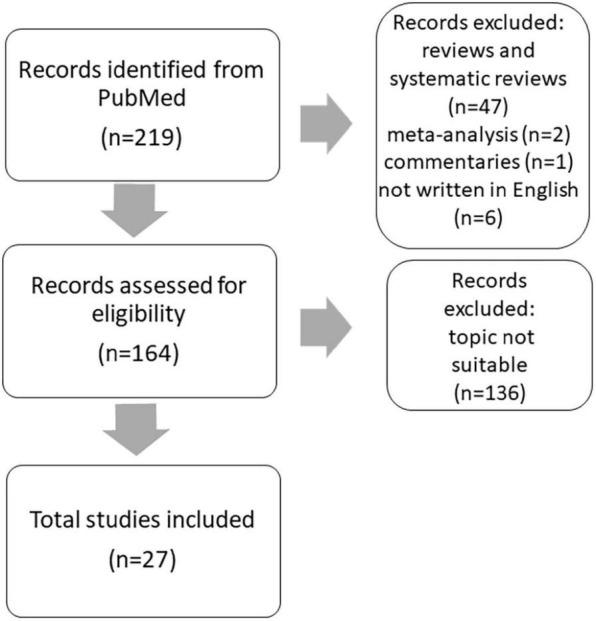
Article inclusion-workflow.

We included 27 papers in total (Supplementary Table 1). The workflow is illustrated in Figure 1. The summary of the psychiatric symptoms including stratification by sex, age and ERT therapy is shown in Table 1.

**TABLE 1:** Neuropsychiatric sympthoms stratified by sex, age, and ERT therapy status.

	Sex	Age	ERT
Cognitive impairment	Males showed impairment in the speed of information processing, reduced performance in executive functions; females were comparable with healthy controls (32, 33). In some studies, no difference between males and females (30)	Children showed limitation in processing and consecutive domains (25)	ERT + group had higher scores on measures of overall cognitive functioning on the PedsQLTM cognitive functional scale, showed lower attention deficits (25)
Schizophrenia	Case reports on both males and females (34–38)	No case reports on children	No case reports
Depression	Mostly no differences between males and females (33, 42)	Not significant depression in children (24)	One case report: an adult patient showed symptom remission (43)
Social functioning	Females had worse social functioning that males (20)	Adolescents had worse social functioning than children (24)	ERT improved social functioning in children (24)

### Neurocognitive Performance

The prevalence of neurocognitive impairment in FD patients (mixed cohorts) differs in various studies from normal cognitive performance (28, 29) to nearly 30% of patients with cognitive impairment (30). Objective cognitive limitations were shown in several domains: especially in executive functioning, information processing speed and attention (16–18) (Supplementary Table 2). However, normal cognitive performance was reported in memory, naming, perceptual functioning, and global cognitive functioning (16–18). Recently, prospective longitudinal studies reported normal cognition at baseline and two follow-ups [2-8 years after the baseline (31)], suggesting a stable disease course. Körver et al. (32) reported no major changes in cognition of 76 FD patients on one-year follow-up. Only four patients showed significant regression in cognitive functioning, however not related to sex, phenotype or stroke. Interestingly, the levels of subjective cognitive complaints were neither related to the objective cognitive performance nor sex and showed association with depressive symptoms (32).

Although most of the studies on cognitive impairment are cohort studies, we also included one case report on a male patient that can shed a light on the molecular mechanisms of cognitive impairment in FD. Okeda and Nisihara (15) presented one of the very few autopsy cases of a patient with FD and early-onset dementia, showing severe neurodegenerative changes *post mortem*. Neuronal ballooning and axonal swelling were most pronounced in the following anatomical structures: the pyramidal tract, pedunculus cerebellaris superior, and brachium colliculi inferioris. Although the vascular component has been described as well, the direct accumulation in the brain tissue resulting in neuronal degeneration suggests pathomechanisms in CNS not related to vasculopathy.

Several risk factors associated with the presence of cognitive impairment have been identified. One of the most important is male sex (OR, 6.8; 95%CI, 1.6-39.8; *p* = 1.6 *10-2) (32). Körver et al. showed that male sex is associated independently with cognitive impairment at baseline, but there were no changes related to sex in neuropsychological assessment after one-year follow-up (31). The study of Sigmundsdottir et al. (33) reported that males with FD had a slower speed of information processing, reduced performance in executive functions (verbal generation, reasoning, problem-solving, perseveration), and they were more likely to show clinically significant limitations. Females with FD performed similarly to healthy controls. Loeb reported the percentage of males and females classified as cognitively intact/impaired was not significantly different (*p* = 0.49) using Fischer’s exact test (30). In contrast, Elstein et al. (16) found the average global cognition scale (GCS) in females lower than in males (90.3 vs. 98.2 points). Higher premorbid intelligence quotient (IQ) (one IQ point increase: OR, 0.91; 95%CI, 0.82-0.98; *p* = 3.8 *10-2) and stroke (OR, 6.4; 95% CI, 1.1-41.0; *p* = 3.7 *10-2) were independently positively associated with more severe cognitive impairment for both males and females (32, 33).

### Neurocognitive Performance in Children

Only a few studies were performed on children with FD. In a mixed cohort composed of adults and children (from 7–61 years), impairment in attention and executive functioning was reported, while intellectual, visual-spatial, language, verbal, and visual memory remained unaffected (17). This finding is consistent with the adult cohorts (32, 33).

In exclusively pediatric cohorts, patients with FD exhibited poorer cognitive and executive functioning in comparison to age-matched controls. The cognitive function of children with FD was comparable to children with head injury (25). Children with FD showed lower scores across various subscales on the Behavior Rating Inventory of Executive Function (BRIEF) in comparison to the healthy controls. FD pediatric patients scored lower on the following scales: GEC (Global Executive Composite) scale, the MI (Metacognition Index) composite scale, and the Initiate, Working Memory, and Organization of Materials subscales. On the BRI (Behavior Regulation Index), Shift and Emotional Control subscales, and Plan/Organize Scale children with FD showed a lower trend in comparison to the healthy controls. The authors concluded that younger age groups may be driving some of the executive functioning findings. This may be related to the fact that older children develop better coping strategies in relation to the disease. No difference between genders has been reported in children. ERT was reported to improve objectively assessed cognitive performance. Children in the ERT + group had higher scores on measures of overall cognitive functioning on the PedsQLTM cognitive functional scale. ERT-treated children also reported fewer symptoms of attention/working memory problems on the BRIEF-MI scale and fewer overall problems of executive functioning, as assessed by the BRIEF-GEC (25).

### Psychosis

Only a few cases of psychosis in FD have been described. Our current knowledge is based on these case reports, but if they are coincidental to FD or not remains unclear (34–38). The studies describing psychiatric symptoms are presented in Supplementary Table 3.

Varela et al. described a family with a mild FD symptom and a novel splice site mutation resulting in two aberrant transcripts (34). The index case’s aunt was diagnosed in the second decade of life with schizophrenia. Segal et al. (17) mentioned briefly that in their FD cohort one patient was diagnosed with schizophrenia, however, no further clinical information was available.

Sawada et al. (35) reported a female patient with FD and delusional and violent behavior at the age of 29. After psychological treatment, the symptoms persisted. At the age of 39, she was readmitted to the psychiatric ward due to the exacerbation of psychiatric symptoms. There was a partial remission after electroconvulsive therapy. Mini-mental state examination (MMSE) was below the norm (20 points).

Gairing et al. (36) reported a 21-year-old female hospitalized for the first time with acute psychotic symptoms. Beforehand, she had suffered from a depressed mood and anxiety for about two years. FD was diagnosed nine years before the psychotic symptoms began. Under the treatment with risperidone and aripiprazole, a partial symptom remission was reached, however, not in the concentration domain. In the battery of neuropsychological tests performed in disease remission, mild cognitive impairments were found in the domain of attention, processing speed, and measures of cognitive flexibility.

In a case report, Shen et al. (37) described a 35-year-old male patient with acute psychotic symptoms. He complained of auditory hallucinations and delusions of reference. FD was diagnosed three years before symptoms began and the patient was regularly receiving ERT. He was initially treated with risperidone, but, due to side effects, switched to aripiprazole and gradual remission was reached. Brain MRI had revealed small hyperintense spots in the right thalamus, midbrain, and corona radiata on T2-weighted imaging, absent in the previous MRI scans. The authors suggested that thalamic lesions can be related to the appearance of schizophrenia symptoms.

The first case of psychosis in an FD was reported by Liston et al. (38). A 26-year old male was admitted to the psychiatric ward with bizarre behavior, paranoid ideation, and auditory hallucinations. Minnesota Multiphasic Personality Inventory (MMPI) suggested schizophrenia or psychotic depressive disorder. FD was diagnosed six years before admission. The symptoms remitted without administration of any psychotic medication.

### Depression

Depression is the most common psychiatric disease among FD patients and contributes to increased morbidity and mortality significantly. The prevalence of depression in FD varies across the studies and reaches up to 60% (18, 32) (Supplementary Table 4). In the largest psychiatric study of FD to date, Cole et al. (19) assessed 184 FD patients with the Center for Epidemiological Studies depression scale (CES-D), a well-validated and widely used questionnaire that assesses current depressive symptoms. They found that 83 (46%) patients met the criteria for clinically significant depressive symptoms, with 50 of those patients (28%) exhibiting severe depressive symptoms. In the study of Segal et al. (17), depression was rarer: the authors reported this diagnosis for four out of 16 patients. Depression was also described in several case reports (39–41). Körver et al. (32, 42) investigated factors associated with CESD-scores and found them lower in patients with a better health perception and more “positivity and problem solving” and higher in patients with more pain and “avoidance and brooding.”

One study showed no significant differences in depression incidence between FD patients and the general population. Sigmundsdottir et al. (33) reported no significant differences between the FD patients and controls assessed with DASS-21 scale (Depression, Anxiety and Stress scale) (depression: *p* = 0.07; anxiety: *p* = 0.06: stress: *p* = 0.61).

Three studies compared depression in males and females and mostly found not diffreneces in terms of sex. In one study (19), unlike in the normal population, depression was more common in males (36%) than in females (22%). In the study of Sigmunsdottir et al. (33) differences for depression scores between male groups approached significance (*p* = 0.05) but, for stress, they did not (*p* = 0.41). Also, Körver et al. (42) found no differences in terms of sex for depression assessed with CESD score. In a pediatric cohort, FD patients were not more severely depressed than the controls, regardless of the age of the patient (24).

Although on the DASS-21 scale for depression, anxiety and stress differences between FD pediatric patients and controls were not significant, there was an improvement according to the other scales and parameters after an administration of ERT. The ERT + group reported significantly lower scores on the attention problems subscale of the BASC-2 (*p* = 0.016) and higher mean scores on the adaptive skill functioning subscale (*p* = 0.012). Parents of children in the ERT + cohort reported lower mean depression subscale scores that approached significance level (*p* = 0.129) (24).

An extensive PubMed search with a variety of keywords identified one case report of an adult patient with an improvement of depression after ERT (43) (this case report is not included in the systematic review). Before starting ERT, the patient was diagnosed with a major depressive disorder (DSM-IV) [Hamilton Depression Scale Score (HDS score) ∼ 31], but refused antidepressants. After the seventh infusion of ERT, the patient was found to be less withdrawn, sad, or anxious. After one year of ERT, the major depressive disorder remitted completely (HDS score ∼ 7).

### Social Functioning and Adaptation

Laney et al. (20) reported impaired social-adaptive functioning, particularly in women with FD. They performed Achenbach’s system of empirically based assessment (ASEBA) on the group of 30 FD patients. ASEBA is a collection of questionnaires used to evaluate adaptive and maladaptive behavior and general functioning. 8/30 FD patients (26.7%) had mean adaptive functioning scores characteristic for social adaptive functioning deficiency (SFAD) on the adult self-report scale.

Interestingly, SFAD was more common among women 6/18 (33%) than men 2/12 (17%). The majority of women with social adapting deficits were moderately affected by FD (4/6); the remaining two were mildly affected at the time of the Fabry-specific Mainz disease severity score (MSS) calculation (20). Only one man in the investigated cohort was described as severely affected by FD.

The study of Bugescu et al. (24) assessed psychosocial functioning in children. The FD group was divided into a childhood (ages 6–11, *n* = 12) and adolescent group (ages 12–18, *n* = 14). Parental reports of adolescents with FD psychosocial functioning showed significantly higher scores on the BASC-2 internalizing problem subscales, including anxiety, depression, and somatization. Younger children with FD had no significant differences compared to a normative group (24). Additionally, the ERT + group showed significantly lower scores on the attention problems subscale of the BASC-2 (*p* = 0.016) and higher mean scores on the adaptive skill functioning subscale (*p* = 0.012) (24).

Not only impaired social-adaptive functioning but also higher attention deficit hyperactivity disorder (ADHD) (24%), inattention (21%) and hyperactivity-impulsivity (21%) were reported in an adult FD sex-balanced cohort (20). FD patients show also some characteristic personality features. A higher prevalence of avoidant personality (36%); antisocial personality (24%); and aggressive behavior (21%) was identified for FD individuals than in the control group. 39% of FD patients had generalized anxiety disorder (20). There was a statistically significant relationship between poorer adaptive function and depression (*p* < 0.01), anxiety (*p* = 0.05), depression and anxiety (*p* = 0.03), antisocial personality (*p* < 0.001), inattention (*p* = 0.03), hyperactivity-impulsivity (*p* < 0.01), and aggressive behavior (*p* = 0.03) (20).

## Discussion

In this systematic review, we analyze literature on neuropsychiatric manifestations in FD available in PubMed database. We investigate the differences in psychiatric manifestations between males and females, adults and children and patients treated with ERT and untreated. Multiple studies reported limitations in cognitive performance and depressed mood, however, the prevalence of those symptoms varies depending on the cohort. In our review, we additionally summarize other than cognitive impairment and depression aspects of the NPS in FD, such as schizophrenia and personality disorders. Further, we stratified our results in terms of age, sex and ERT therapy. The FD participants showed a variety of symptoms, from being clinically normal to severe psychiatric manifestations. The discrepancies in assessing NPS between studies can be attributed to the different scales used and to the differences between the cohorts. In the cognitive domains, limitations were reported mainly in attention, psychomotor speed, and executive functions. Males seem to be more severely affected than females in terms of cognitive impairment, however not in terms of depression. Interestingly, FD patients showed also deficits in social-adaptive functioning, more common among women. Adolescents reported impaired social functioning, that however was not observed among younger children. Based on the available studies, ERT alleviates the psychiatric manifestations of FD. The differences between males vs. females, children vs. adults and ERT + vs. ERT – patients are summarized in Table 1.

In recent years, several longitudinal studies on NPS in FD have been performed. They demonstrated either cognitive stability after 8 years (29) or reported no major changes after 1-year (31, 32). The course of depression measured with the Centre for Epidemiological Studies – Depression (CESD) scale was also stable on follow-up (31, 32). No difference on the follow-up has been observed between males and fameles in terms of cognitive performance.

Only few reviews and systematic reviews assessing neuropsychiatric aspects of FD have been published so far (42, 44–46). They highlighted cognitive impairment in executive functioning, information processing speed, and attention, while other domains, such as general intellectual functioning, memory, naming, perceptual functioning, and global cognitive functioning remained intact. The prevalence of depression varied from 15 to 62%, and neuropathic pain was recognized as a main contributing factor (46). A review article on depression in FD (46) highlighted the influence of pain on the depressive symptoms, but questioned the relation between cerebrovascular disease, imaging parameters and depression. To our best knowledge there is no review article highlighting impact of sex, age or ERT status on NPS in FD. The reason for it can be that there are still few data on these issues.

Several limitations of this systematic review need to be acknowledged. First of all, psychiatric evaluation of the FD patients is not a medical routine yet, so that only FD patients more severely affected and showing psychiatric abnormalities may be referred to the psychiatrist and seek professional help. This can be especially the case for females that are often undiagnosed and only those with psychiatric symptoms may be included in the psychiatric observational studies. Also, the outcome of the study may be influenced by the usage of different scales. A broad range of scales has been applied so far, but the standardized test battery is lacking. This can explain different outcomes of the several studies. Another problem is the small size of the cohorts. Only a fraction of studies perfomed a stratification in terms of age and sex. Additionaly FD is a rare disease with a frequency typical presentation of around 1 in 22,000 to 40,000 in males and unknown frequency in women (47). Small cohorts contribute to the fact that results often do not reach statistical significance. However, as with any rare disease, small sample size is likely to be a limitation in future investigations. For some disease-modifying factors, such as ERT therapy in depressed and cognitively impaired adults, only a few case reports are available so that no conclusions can be drawn.

### Future Research Fields

We would like to highlight three future research areas: investigating molecular disease mechanisms, brain imaging and diagnostic and therapy (Supplementary Table 5). Molecular mechanisms and medical imaging can also identify screening, prognostic and pharmacodynamic biomarkers.

The molecular mechanism of neuropsychiatric symptoms in FD is still not fully understood. The pathomechanism is mostly attributed to the changes in small vessels related to the accumulation of Gb3, small vessel stroke, and dilated vasculopathy. Other mechanisms of brain involvement in FD are suspected recently, especially those associated with neuroinflammation, and activation of molecular pathways related to neurodegeneration and direct deposition of GL-3 in brain tissue, especially in brain regions associated with neuropsychiatric symptoms. An interesting mechanism connecting direct nerve tissue damage and vascular degeneration and based on the *vasa vasorum* impaired perfusion has been described recently (48).

Three components of brain involvement related to stroke are to be recognized, namely endothelial cell dysfunction, impaired vessel wall structure and function, and altered blood components. Preclinical data suggest that deregulation of key endothelial pathways (23). Increased oxidation stress attributed to the NOS secretion and production of the free radicals can cause vessel dilatation leading to changes in perfusion (49). Other molecular mechanisms, such as inflammation and altered gene expression, may play a role in the development of neuropsychiatric symptoms (50–52). ERT supplementation reduced inflammation markers in the musrine FD model (53).

The second research area that gained importance in recent years is brain imaging. Characteristic neuroimaging features are diffuse white matter lesions (WML) and stroke related to the small vessel’s disease (10). Although FD patients present with a WML, compared to the multiple sclerosis, they show a very low incidence of corpus callosum involvement (54). Another radiological feature common for FD patients is a pulvinar sign, definded as exclusive T1WI hyperintensity of the lateral pulvinar. Although is it easy to identify, its incidence is lower as previously expected (around 3%) (55). Cognitive dysfunction can mainly be related on the radiological level to hippocampal atrophy, decreased brain volume, abnormalities in diffusion tensor imaging and compensatory functional alterations (56). One case report suggests a direct link between anatomical Gb3 deposition and the schizophrenia. FD patient with psychosis showed small hyperintense spots in the right thalamus, midbrain, and corona radiata on T2-weighted imaging brain MRI, regions involved in the pathogenesis of psychotic symptoms (37).

The third area of research is to improve the diagnosis of NPS in FD and treatment. Although cognitive impairment and depression are symptoms most frequently occurring in FD, patients should be also observed in the context of social activities, adaptation skills, and psychotic symptoms. Comprehensive treatment plans should consider therapies to improve occupational and psychological functioning, which could lead to improved treatment compliance, daily functioning, and better quality of life and showed to be effective in depression (57). Considered a great impact of pain in depressed FD individuals, a certain antidepressant, such as the dual serotonin and noradrenaline reuptake inhibitors, targeting both depression and pain, are of interest and may allow treating both disorders with a single medication (46). Finally, of special interest is the assessment of ERT impact on NPS. Until now, the influence of ERT has been described only in pediatric cohorts in terms of standardized clinical studies, but studies on adult cohorts are expected.

Neuropsychiatric symptoms in FD often remain undiagnosed. FD patients present not only with cognitive impact and depression, but also with impaired psychosocial functioning and characteristic personality features. Several cases of schizophrenia have been described, although they may not be directly related to FD. The healthcare practitioners should be aware of the psychiatric manifestations and their prevalence in FD patients and offer both psychological support and/or medication to their FD patients. Females and children/adolescent should be screened for psychiatric manifestations as well. ERT therapy seems to partially restore cognitive function impairment and alleviate other neuropsychiatric symptoms. Comprehensive treatment plans should consider therapies to improve social, occupational, and psychological functioning. Through our review, we would like to to raise awarness of neuropsychiatric symptoms in FD for the general public, including general practitioners and all medical doctors involved in the care of FD patients and highlight the necessity to screen also females, children and individuals undergoing ERT.

## Author Contributions

MM, IM, and AN contributed to conception and design of the study. ES, RB, and MS edited the mansucript and wrote sections of the mansucript. MM and IM wrote the first draft of the manuscript. All authors contributed to manuscript revision, read, and approved the submitted version.

## Conflict of Interest

AN received research support and speaker honoraria from Amicus, Takeda and Sanofi Genzyme. RB declares Grant/Research Support from: Pfizer Pharma GmbH, Genzyme GmbH, Grünenthal GmbH, Mundipharma Research GmbH und Co. KG., Novartis Pharma GmbH, Alnylam Pharmaceuticals Inc., Zambon GmbH, Sanofi-Aventis Deutschland GmbH. Speaker: Pfizer Pharma GmbH, Genzyme GmbH, Grünenthal GmbH, Mundipharma, Sanofi Pasteur, Medtronic Inc. Neuromodulation, Eisai Co. Ltd., Lilly GmbH, Boehringer Ingelheim Pharma GmbH&Co., KG, Astellas Pharma GmbH, Desitin Arzneimittel GmbH, Teva GmbH, Bayer-Schering, MSD GmbH, Seqirus Australia Pty. Ltd., Novartis Pharma GmbH, TAD Pharma GmbH, Grünenthal SA Portugal, Sanofi-Aventis Deutschland GmbH, Agentur Brigitte Süss, Grünenthal Pharma AG Schweiz, Grünenthal B.V. Niederlande, Evapharma, Takeda Pharmaceuticals International AG Schweiz, Ology Medical Education Netherlands, Ever Pharma GmbH. He also declares consultancy work for: Pfizer Pharma GmbH, Genzyme GmbH, Grünenthal GmbH, Mundipharma Research GmbH und Co., KG, Allergan, Sanofi Pasteur, Medtronic, Eisai, Lilly GmbH, Boehringer Ingelheim Pharma GmbH&Co., KG, Astellas Pharma GmbH, Novartis Pharma GmbH, Bristol-Myers Squibb, Biogenidec, AstraZeneca GmbH, Merck, Abbvie, Daiichi Sankyo, Glenmark Pharmaceuticals S.A., Seqirus Australia Pty. Ltd., Teva Pharmaceuticals Europe Niederlande, Teva GmbH, Genentech, Mundipharma International Ltd., United Kingdom, Astellas Pharma Ltd., United Kingdom, Galapagos NV, Kyowa Kirin GmbH, Vertex Pharmaceuticals Inc., Biotest AG, Celgene GmbH, Desitin Arzneimittel GmbH, Regeneron Pharmaceuticals Inc., United States, Theranexus DSV CEA Frankreich, Abbott Products Operations AG Schweiz, Bayer AG, Grünenthal Pharma AG Schweiz, Mundipharma Research Ltd., United Kingdom, Akcea Therapeutics Germany GmbH, Asahi Kasei Pharma Corporation, AbbVie Deutschland GmbH&Co., KG, Air Liquide Sante International Frankreich, Alnylam Germany GmbH, Lateral Pharma Pty Ltd., Hexal AG, Angelini, Janssen, SIMR Biotech Pty Ltd., Australien, Confo Therapeutics N. V. Belgium, Merz Pharmaceuticals GmbH, Neumentum Inc., F. Hoffmann-La Roche Ltd., Switzerland. MS has received personal fees from Sanofi Genzyme, Grünenthal GmbH, Amicus Therapeutics and Takeda Pharmaceutical, outside the submitted work. ES received in the last 3 years honoraria and grants for advice and educational lectures from Lundbeck Switzerland, Schwabe Switzerland and Germany, Janssen Switzerland, Otsuka Switzerland, Mepha Pharma Switzerland, Otsuka Pharma Switzerland, Ricordati Switzerland and Sunovion Pharma UK and Angelini. The remaining authors declare that the research was conducted in the absence of any commercial or financial relationships that could be construed as a potential conflict of interest.

## Publisher’s Note

All claims expressed in this article are solely those of the authors and do not necessarily represent those of their affiliated organizations, or those of the publisher, the editors and the reviewers. Any product that may be evaluated in this article, or claim that may be made by its manufacturer, is not guaranteed or endorsed by the publisher.
