# Supplementary material for: Neuropsychiatric Symptoms and Their Association With Sex, Age, and Enzyme Replacement Therapy in Fabry Disease: A Systematic Review
Source: Front Psychiatry. 2022 Mar 16;13:829128. doi: 10.3389/fpsyt.2022.829128 (PMC8967288; doi:10.3389/fpsyt.2022.829128)
Supplement: Supplementary file 1 [file Data_Sheet_1.docx]

**Supplementary Table 1. Clinical studies and case reports included in the review**

| **Study type** | **Cohort** | **Reference** |
| --- | --- | --- |
| Case report | 4 patients | [34] |
| Longitudinal | 13 patients | [39] |
| Longitudinal | 81 patients | [31] |
| Longitudinal | 81 patients | [32] |
| Cross-sectional | 37 patients | [58] |
| Cross-sectional | 81 patients | [42] |
| Cross-sectional | 41 patients | [30] |
| Case report | 1 patient | [39] |
| Cross-sectional | 24 patients | [24] |
| Case reports | 1 patient | [35] |
| Longitudinal | 14 patients | [29] |
| Cross-sectional | 110 patients | [59] |
| Cross-sectional | 24 patients | [25] |
| Cross-sectional | 12 patients | [33] |
| Cross-sectional | 10 patients | [16] |
| Case report | 1 patient | [36] |
| Cross-sectional | 25 patients | [18] |
| Cross-sectional | 16 patients | [17] |
| Cross-sectional | 33 patients | [20] |
| Case report | 1 patient | [15] |
| Cross-sectional | 184 patients | [19] |
| Case report | 1 patient | [35] |
| Case report | 1 patient | [38] |
| Cross-sectional | 4 patients | [41] |
| Case report | 1 patient | [60] |
| Retrospective | 6 patients | [61] |
| Case report | 1 patient | [38] |

**Supplementary Table 2. Studies on neurocognitive performance**

| **Tests** | **Cohort** | **Result summary** | **Reference** |
| --- | --- | --- | --- |
| Digit span (WAIS-III) test, Trail Making test (TMT), Stroop Color Word Test (SCWT), Rey Auditory Verbal Learning Test (AVLT), Rey Visual Design Learning Test (VDLT), Picture Completion test (WAIS-III), the Block Design test (WAISIII) Judgement of Line, Digit Symbol coding test, controlled Oral Word Test, The Similarities (WAIS-III), Arithmetic (WAIS-III) and the Boston Naming Test | 13 patients at baseline, 6 at the first follow-up, 4 at the second follow-up | Normal scores on all tests at baseline and during follow-up | [28] |
| 16 subtests representing the following cognitive domains: language, memory, visuospatial perception, attention and executive functioning and processing speed; different test used at baseline and follow-up to avoid learning effect | 81 patients, 73 controls at baseline | 4 patients (5.3%) showed reliable decrease in cognitive functioning, 2 women and 1 man with classical disease and 1 woman with non‐classical disease (age range: 19‐41 years). Changes were from excellent to good/average and from good to average. | [31,32] |
| A battery of test measuring language, memory, visuospatial perception, attention and executive functioning and processing speed | 81 patients | Subjective cognitive complaints were reported by 64% of all patients. Objective cognitive impairment was present in thirteen patients (16%). Male sex, stroke positively associated and premorbid IQ negatively associated. | [58] |
| Neuropsychological tests (17 measures) and evaluation of subjective complaints with the Perceived Deficits Questionnaire (PDQ) | 41 patients | 12 patients (29.3%) had a cognitive impairment. Tests tapping psychomotor speed, attention and executive functions had the highest frequency of impairment. | [30] |
| Rey Auditory Verbal Learning Task (AVLT), Wechsler Memory Scale Revised (WMS-R), Trail Making Test A and B (TMT-A and-B), MMSE, HAMD-17 | 25 patients at baseline and 13 at follow up | No statistically differences in cognitive performance between baseline and follow up after 8 years | [29] |
| The Behaviour Rating Inventory of Executive Function (BRIEF), Peds QL cognitive functioning scale | 24 paediatric FD patients | Lower overall cognitive functioning on the PedsQLTM CFS, children with FD showed lower scores across various composite scales and subscales on the BRIEF score | [25] |
| Extensive neuropsychological testing assessing ten cognitive domains, including intellectual functioning (Test of Premorbid Functioning, Wechsler Adult Intelligence Scale, Fourth Edition; WAIS-IV), memory (California Verbal Learning Test, Second Edition; CVLT-II; Wechsler Memory Scale, Fourth Edition), immediate attention and working memory (WAIS-IV Digit Span), visuospatial (WAIS-IV Block Design), speed of information processing (WAIS-IV Coding and Symbol Search), language (WAIS-IV Vocabulary, Boston Naming Test), reasoning skills (WAIS-IV Similarities, Matrix Reasoning and Visual Puzzles), verbal fluency (Verbal Fluency), and problem solving (Wisconsin Card Sorting Test [WCST]) | 17 FD patients | Significant differences were found between male FD participants and male controls on general intellectual functioning, speed of information processing, reasoning, verbal fluency and problem solving/perseveration | [33] |
| Mindstreams neurocognitive battery for mild impairment, evaluating memory, executive function, attention, information processing, visual spatial processing, verbal function, and motor skills, Global cognitive scores | 10 patients | 3 females had mild-moderate neurological MSS subscores (two CVA); all females had Mindstreams GCS of 59-107.7 points. Below-average performance was reported particularly in information processing and motor skills consistent with mild impairment. Average GCS in females (90.3 points) was lower than in males (98.2 points). | [19] |
| Rey Auditory Verbal Memory Test (AVLT), Visual Reproduction of the Wechsler Memory Scale (WMS-R), computerized attention test battery TAP, Trail Making Test (TMT) A and B, Wisconsin Card Sorting Test | 25 FD patients | Patients with FD showed deficits only in the attention domain. | [18] |
| Wechsler Adult Intelligence Scale, The Rey-Osterrieth Complex Figure Test (ROCF), Kave Naming Test (KNT)Choice reaction time tests, Motor tapping tests, Integneuro CPT test, first part of the switching of attention test -digits only, the first part of the verbal interference test (a computerized version of the Stroop test)—word only and the digit symbol subtest from the WAIS-III or WISC-R95, Switching of attention (second part, letters and digits), Verbal interference (second part—color), digit span backward and the maze test. | 16 FD patients | Performance on most cognitive measures was within the average range. All measures of information processing speed were significantly reduced, as were some measures of executive functions. | [17] |

**Supplementary Table 3. Case reports of psychosis and FD**

| **Main psychiatric symptoms** | **Treatment of psychiatric symptoms** | **Age at onset** | **Other symptoms of FD** | **Reference** |
| --- | --- | --- | --- | --- |
| Hallucinatory, delusional state after delivery of her daughter, delusional and violent behaviour | Psychotherapy, electroconvulsive therapy | First symptoms at 27 years, exacerbation at the age of 39 | Recurrent fever, foot numbness (42 years old) | [35] |
| Delusions of reference, persecutory delusions and auditory hallucinations. | Risperidone (not tolerated in the dose 2 mg), remission under aripiprazole (10 mg) | First psychotic symptoms at 21 years; depressed mood, anxiety since 14 years of age | Pain, acroparesthesia, hypohydrosis, abdominal pain, postprandial diarrhoea and fatigue, 9 sibs affected by FD, one with schizophrenia | [36] |
| Auditory hallucinations and delusions of reference, thought processes were loose and disorganized and there was evidence of thought blocking. | Risperidone at 1 mg showed side effects, remission under aripiprazole 5 mg | 35-years | Pain, acroparesthesia | [37] |
| Bizarre behaviour, paranoid ideation, auditory hallucination  and episodic impairment of memory | Phenoxybenzamine increased to 3 x 10 mg, recurrence in one year, treatment with phenoxybenzamine led again to the remission | 21 years | Extremity pain | [38] |

**Supplementary Table 4. Studies on depression**

| **Test** | **Cohort** | **Result summary** | **Reference** |
| --- | --- | --- | --- |
| CESD | 81 patients, 73 controls at baseline | At baseline, 29 patients (38.2%) scored ≥16 on the CESD and 22 patients (28.9%) had a history of depressive disorder between baseline and follow‐up, 6 patients diagnosed with depressive disorder (7.9%). | [31] |
| CESD | 81 patients | CESD-scores were lower in patients with better health perception and more "positivity and problem solving" and higher in patients with more pain and "avoidance and brooding". | [32] |
| Hamilton Depression Rating Scale (HAM-D) 21 | 37 patients | HAM-D indicated depression in 21 patients (56.8%). Twelve patients were classified as mild; 4 patients as moderate, and 5 patients as having severe depression. | [58] |
| CDI-2 | 24 paediatric patients | CDI-2 did not show significant differences, 21% of patients reported levels of depressive symptomatology within the clinical range | [25] |
| HAMD-17, Positive and Negative Syndrome Scale (PANSS) | 25 FD patients | Clinically relevant depressive syndromes were noted in 60% of the patients. | [18] |
| SADS/K-SADS | 16 FD patients | reduced performance on 3 out of the 8 tests of executive functions. | [17] |
| CES-D | 184 patients | The prevalence of depression was 46%, of which 28% were consistent with severe clinical depression. | [19] |
| DSM criteria | 33 patients | 5 suffered depression, two of them committed suicide. | [20] |

**Supplementary Table 5. Summary of future research fields**

| **Disease mechanism** | **Imaging** | **Diagnosis and therapy** |
| --- | --- | --- |
| - Endothelial pathways activation; - Free radicals; - Inflammation; - Molecular markers. | - Correlation of neuroimaging features with neuropsychiatric symptoms; - Anatomical deposition of Gl3 in strategic brain regions; - Neuroimaging progression and outcome markers. | - Standardized test battery for screening and follow up; including neuropsychiatric screening for the standard assessement of FD patients; - Longitudinal studies; - Psychotherapeutic and occupational therapies; - Pharmacological treatment tailored for FD patients; - Assessing impact of ERT on neuropsychiatric symptoms. |

References:

1. Rosa Neto NS, Bento JCB, Pereira RMR. Depression, sleep disturbances, pain, disability and quality of LIFE in Brazilian Fabry disease patients. Mol Genet Metab Rep (2019) Dec 2;22:100547. doi: 10.1016/j.ymgmr.2019.100547
2. Löhle M, Hughes D, Milligan A, Richfield L, Reichmann H, Mehta A, Schapira AH. Clinical prodromes of neurodegeneration in Anderson-Fabry disease. Neurology (2015) Apr 7;84(14):1454-64. doi: 10.1212/WNL.0000000000001450.
3. Mendez MF, Stanley TM, Medel NM, Li Z, Tedesco DT. The vascular dementia of Fabry's disease. Dement Geriatr Cogn Disord (1997) Jul-Aug;8(4):252-7. Doi: 10.1159/000106640.
4. Grewal RP. Psychiatric disorders in patients with Fabry’sdisease. Int J Psychiatry Med (1993) 23: 307–312. doi: 10.2190/JKFW-3WXK-QA7N-BYLN
